# Role of Nuclear Factor Erythroid 2 (Nrf2) in the Recovery of Long COVID-19 Using Natural Antioxidants: A Systematic Review

**DOI:** 10.3390/antiox11081551

**Published:** 2022-08-10

**Authors:** Muchtaridi Muchtaridi, Siti Rafa Amirah, Jacko Abiwaqash Harmonis, Emmy Hainida Khairul Ikram

**Affiliations:** 1 Department of Pharmaceutical Analysis and Medicinal Chemistry, Faculty of Pharmacy, Universitas Padjadjaran, Jl. Raya Bandung-Sumedang KM 21, Sumedang 45363, Indonesia; 2 Faculty of Health Sciences, Universiti Teknologi MARA, Bandar Puncak Alam 42300, Malaysia

**Keywords:** long COVID-19, Keap1, Nrf2, natural antioxidant

## Abstract

Coronavirus disease 2019 (COVID-19) is an infectious disease with approximately 517 million confirmed cases, with the average number of cases revealing that patients recover immediately without hospitalization. However, several other cases found that patients still experience various symptoms after 3–12 weeks, which is known as a long COVID syndrome. Severe acute respiratory syndrome coronavirus 2 (SARS-CoV-2) infection can activate nuclear factor kappa beta (NF-κβ) and unbind the nuclear factor erythroid 2-related factor 2 (Nrf2) with Kelch-like ECH-associated protein 1 (Keap1), causing inhibition of Nrf2, which has an important role in antioxidant response and redox homeostasis. Disrupting the Keap1–Nrf2 pathway enhances Nrf2 activity, and has been identified as a vital approach for the prevention of oxidative stress and inflammation. Hence, natural antioxidants from various sources have been identified as a promising strategy to prevent oxidative stress, which plays a role in reducing the long COVID-19 symptoms. Oxygen-rich natural antioxidant compounds provide an effective Nrf2 activation effect that interact with the conserved amino acid residues in the Keap1-binding pocket, such as Ser602, Ser363, Ser508, and Ser555. In this review, the benefits of various natural antioxidant compounds that can modulate the Nrf2 signaling pathway, which is critical in reducing and curing long COVID-19, are highlighted and discussed.

## 1. Introduction

The coronavirus disease 2019 (COVID-19) pandemic, caused by severe acute respiratory syndrome coronavirus 2 (SARS-CoV-2) infection, has become a global health problem worldwide [1]. Moreover, along with its development, SARS-CoV-2 mutated into various variants, such as Alpha, Beta, Gamma, Delta, Omicron, Lambda, and Mu, drawing the world’s attention. Almost all these variants where data were available showed mild symptoms, such as anosmia, cough, flu, sore throat, and fatigue. Even the Omicron variant causes milder symptoms and is generally asymptomatic, one of which is because there is a protein structure in the virus that does not undergo mutations so that it can induce the mechanism of action of the immune system of infected patients [2].

Various fields, including modern health, medicine, economy, and society, are affected by this pandemic. According to World Health Organization (WHO) data, as of 13 May 2022, there were 517,648,631 confirmed cases of COVID-19, including 6,261,708 deaths. With 60% of those infected recovering after 28 days without hospital treatment, most patients still experience various symptoms after 3–12 weeks, known as the long COVID syndrome [1,3].

Regardless of the virus variant, one of the causes of long COVID is due to the impaired expression of antioxidant enzymes and cytoprotective proteins that are regulated by the response of antioxidant elements to deoxyribonucleic acid (DNA), resulting in oxidative stress. In this phase, the transcription factor nuclear factor erythroid 2-related factor 2 (Nrf2) and the formation of the inflammasome have an important role in the occurrence of long COVID-19 [4]. In addition, long COVID-19 may also be caused by a combination of direct harm from SARSCoV-2, immunological activation, mental and emotional factors, and comorbidities.

Antioxidants are substances that can delay, slow down, or prevent the oxidation process, and are therefore important to the body. Naturally, our body has a defense mechanism against oxidative stress through endogenous antioxidants. Consequently, when the number of free radicals and reactive species in the body is out of balance and exceeds the ability of endogenous antioxidants, antioxidants obtained from food or drugs are required [5].

Antioxidant compounds can be either natural or synthetic compounds. Currently, synthetic antioxidant compounds are not preferred due to studies indicating possible adverse health effects, thus natural antioxidants are in demand because of their better safety. Noteworthy, natural antioxidants have many benefits ranging from health and beauty to food [6].

In the food and health sectors, various natural antioxidants have shown promising potential for antioxidant response and Nrf2 translocation [7,8]. Both exogenous and endogenous natural antioxidant compounds have the potential to reduce cytokine storm, endothelial damage, insulin resistance, lung injury, and others [9]. Therefore, this review focuses on the prospect of utilizing natural antioxidants as long-term treatment for COVID-19 via the Nrf2 signaling pathway. This would be useful for researchers to conduct further research on the activity of natural antioxidant compounds that could be used as long-term therapeutic targets for COVID-19.

## 2. Materials and Methods

**Figure 1:**
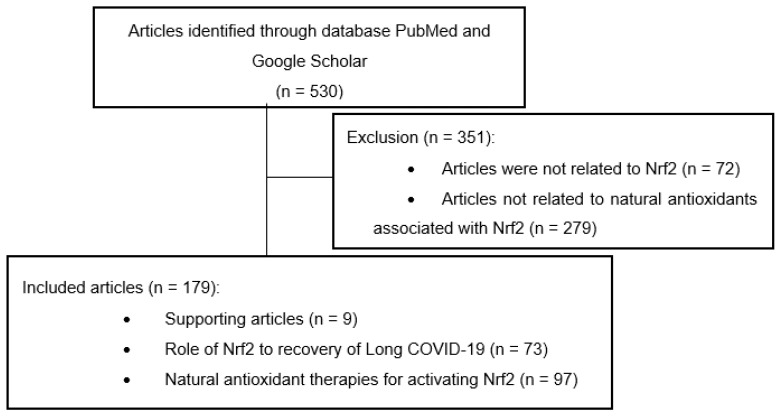
Literature search flow chart.

This systematic review is designed based on a literature search on the PubMed and Google Scholar databases by listing seven keywords, namely, “Nrf2”, “Long COVID-19”, “Natural Antioxidants”, “Role of Nuclear Factor Erythroid 2 (Nrf2)”, “Nrf2 AND Long COVID-19”, “Nrf2 AND Natural Antioxidants”, and “Nrf2 AND Long COVID-19 AND Natural Antioxidants”. Relevant titles and literature abstracts were then sorted, and the full text was examined according to the inclusion criteria. The inclusion criteria were articles reporting on the natural pharmacological activity of antioxidants in the Nrf2 signaling pathway against long COVID. In addition, tracing back was carried out from the list of relevant references of the selected papers. The flow of the article search is shown in Figure 1.

## 3. Long COVID

Long COVID or persistent COVID is a heterogeneous group that experiences long-term persistent symptoms after suffering from the acute phase of COVID-19 [10]. The recent emergence of a number of people experiencing symptoms of long COVID-19 means that there is no standardized framework for identifying and assessing the associated symptoms or other clinical indicators. Some of the proposed frameworks are made without clear criteria on how to define conditions and stratification of patients, resulting in ambiguity in much of the data in the paper; indeed, these papers do not fit the single definition of long COVID and do not evaluate consistent symptoms or markers of the disorder [11]. Most studies also do not assign patients to a specific diagnosis or operational definition, although some refer to the definition from Greenhalgh et al. (2020), which is consistent with the virological data available so far [12].

In a study conducted by Rando et al. (2021), most studies use a survey-based approach, and some studies have used imaging and other technologies to identify physical signs of organ damage. While heterogeneity in the presentation of Long COVID has been identified, the specific variables affecting outcomes remain to be characterized [11].

Post-COVID syndrome can occur continuously or recur and go into remission [13]. These symptoms affect multiple organ systems, including the nervous system (headache, difficulty concentrating), the breathing tract (chest pain, cough, shortness of breath), pathological inflammation (immune dysregulation, autoimmunity, and viral persistence), and muscles and joints (myalgia and arthralgia). In addition, non-specific symptoms, such as fatigue (the most frequently reported symptom) and hair loss, may be apparent. However, this syndrome remains unknown due to the lack of diagnostic criteria for coronavirus symptoms from the persistent period following the acute phase. The cause of the symptoms is still unknown, but it is most likely due to a combination of direct harm from SARS-CoV-2, immunological activation, comorbidities, and mental and emotional factors [12].

**Figure 2:**
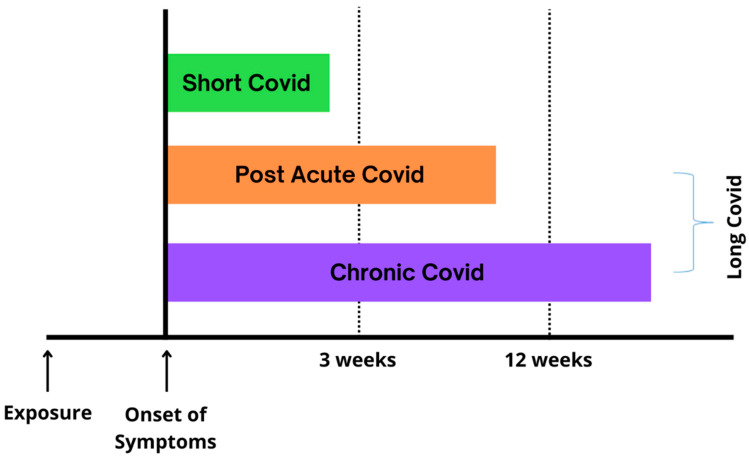
Classification of long COVID.

The post-COVID or COVID period can be classified into two stages, namely, post-acute COVID and chronic COVID, as shown in Figure 2. Post-acute COVID symptoms can last from 3 to 12 weeks. Meanwhile, chronic COVID symptoms can last longer, which is more than 12 weeks [12]. It is also known that the risk factors for long COVID are gender, smoking, and chronic obstructive pulmonary disease (COPD). However, this risk factor is still ambiguous and needs further research because there are no consistent data so far. This may be due to several symptoms and pathophysiologies, ranging from long-term damage to multiple organ systems to persistent inflammation from multiple sources [14].

Studies have shown that symptoms of long COVID may be related to a pathophysiology beyond lung injury, such as persistent neurological complications. For example, structural and metabolic abnormalities of the brain were reported after three months of treatment, correlating with the symptoms of persistent neurological complications such as anemia, fatigue, and memory loss [15,16]. This suggests that most people with mild COVID-19 can have a lasting and resistant effect on the brain. Hence, the severity of COVID-19 plays a small role in predicting this neurological problem [16,17].

A meta-analysis inspecting neuropsychiatric results in sufferers of Middle East Respiratory Syndrome (MERS), Severe Acute Respiratory Syndrome (SARS), and COVID-19 has discovered delirium to be a common complication in the intense segment of the illness, with the neuropsychiatric signs consisting of anxiety, depression, post-demanding pressure disorder, fatigue, and memory loss [17,18]. Other evidence reported that long COVID is also present in patients with cardiac injury, which is now no longer related to preliminary COVID-19 severity. Symptoms of heart problems consisting of palpitations, tachycardia, and chest pain normally persist for up to 6 months, suggesting enormous cardiac sequelae. This was obtained from the results of a multivariable analysis that examined the relationship between disease severity and categorical outcomes [18,19,20,21].

A study reported that in 50% of asymptomatic COVID-19 cases, the nucleic acid and protein of SARS CoV-2 persist in the small intestine after 4 months of the post-disease phase [22]. The study explained that the presence of SARS-CoV-2 can become persistent in the body causing some level of immune activation. The overview shows that T cell dysfunction may also promote comparable long COVID pathophysiology in autoimmune diseases such as systemic lupus erythematosus (SLE) and rheumatoid arthritis [23,24,25,26]. Between 15 and 20% of the COVID-19 patients were detected to have thyroid dysfunction, which is closely related to T cell mediation [27,28]. Hence, thyroid dysfunction is also one of the pathophysiological factors of autoimmunity in long COVID [27,29]. In addition, B cells also need to be monitored for the autoimmunity of long COVID.

In addition, there is evidence that severe COVID-19 can cause lymphopenia (deficiency of B and T cells), which eventually leads to an inflammatory process. In severe COVID-19 patients, the cause of lymphopenia is likely due to increased cytokine levels and decreased T cell numbers due to SARS-CoV-2 virus infection, which can cause T cell exhaustion and infect and interfere with T cell expansion; however, patients with severe COVID-19 conditions tend to have low lymphocyte counts [30,31].

Indeed, lymphocytes, mainly T cells, play a role in the resolution of post-infection inflammation [32,33]. When T-cell and B-cell lymphocytes are renewed, there will be a long COVID predisposition in the presence of unresolved hyperinflammation [31,33]. Decreases in the number of B cells and T cells are also associated with the continued release of SARS-CoV-2, which will prolong chronic immune activation over the long COVID period of pathogenesis [34,35]. A study has shown that increased levels of vascular-associated proinflammatory biomarkers demonstrated a close association with lung damage in patients who developed COVID-19 after three months of discharge [36]. In contrast, some studies argued that the unresolved inflammation which may only partially explain the pathophysiology of long COVID, with the possibility that the symptoms are associated with other inflammation such as joint pain, muscle pain, and fatigue due to ROS [37,38].

Another possible source of the unresolved inflammation in long COVID could lie in the gut. SARS-CoV-2 replicates efficiently in gastric and intestinal cells, as overexpression of angiotensin-converting enzyme 2 (ACE2) receptors leads to increased fecal excretion in patients with SARS-CoV-2 [39,40,41]. It has been reported that one-third of patients with long-term COVID-19 have problems with gastrointestinal symptoms [21,42]. Thus, it is also concluded that the persistence of SARS-CoV-2 in the digestive tract is the underlying reason for the gastrointestinal manifestations of long COVID.

**Figure 3:**
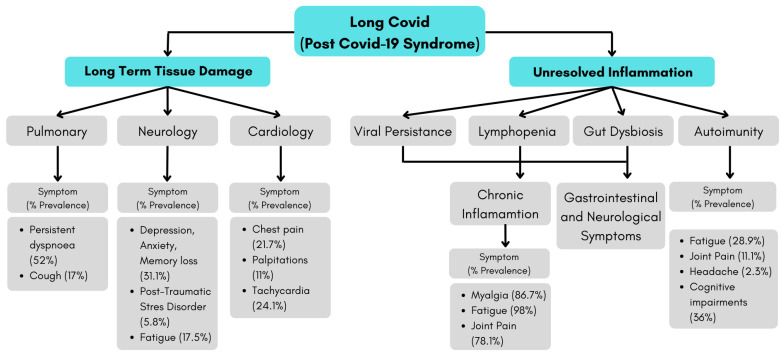
Putative pathophysiology and symptoms of long COVID.

The pathophysiology that causes long COVID is shown in Figure 3. Along with the prevalence that occurs in each symptom [18,19,43,44,45,46,47,48,49,50].

A study conducted on people who had the ABO blood type and then underwent a SARS-CoV-2 test to determine the influence of ABO and rhesus (Rh) blood types on the risk of SARS-CoV-2 infection and more severe COVID-19 disease, showed that people who have blood type O and rhesus-negative (Rh−) status have a slightly lower risk of SARS-CoV-2 infection and severe COVID-19 disease. This is because blood types O and Rh− are protective against severe SARS-CoV-2 infection [51]. In addition, there is a close association between regulation of immune-effector processes and negative regulation of NIK/NF-kappa B signaling against several microRNAs (miRNAs) and long noncoding RNAs (lncRNAs) that are differentially expressed in peripheral blood samples of COVID-19 patients [52].

## 4. Oxidative Stress Associated with SARS-CoV-2 Infection

Reactive oxygen species (ROS) are a group of metabolites, derived from molecular oxygen (O_2_), which are usually reduced by single- or double-electron mechanisms [53], producing superoxide or hydrogen peroxide, respectively, which cause oxidative stress. ROS can be generated from exogenous sources, such as ionizing radiation or redox cycle xenobiotics, and endogenously, as a by-product of aerobic metabolism, with mitochondria as the main source of ROS and being the central regulator of aerobic energy production [54,55]. Within the mitochondria, there are dominant ROS buffer systems, including the glutaredoxin (Grx), glutathione (GSH), and thioredoxin (Trx) systems. In the matrix, the dismutation of O_2_^−^ to hydrogen peroxide (H_2_O_2_) occurs via superoxide dismutase (SOD)2 (MnSOD), while in the intermembrane space the dismutation is carried out by SOD1 (Cu, Zn-SOD). In addition, the breakdown of H_2_O_2_ into O_2_ and H_2_O through the GSH redox system includes glutathione reductase, glutathione peroxidase (GPX), and peroxiredoxins (Prdxs) [54].

The pentose phosphate pathway (PPP) is a minor pathway of glucose metabolism in the resting brain. However, PPP can be regulated under various circumstances to produce NADPH, which then increases GSH and then together with glutathione peroxidase detoxifies ROS [56].

Historically, after ROS were discovered in biological materials, Denham Harman hypothesized that oxygen radicals were formed as a by-product of enzymatic reactions in vivo. Free radicals were described as “a Pandora’s box of evils” that can cause damage, mutagenesis, cancer, and degeneration in aging. McCord and Fridovich also discovered the enzyme SOD, and convinced many people that ROS play an important role in biological processes [56].

The body must maintain the level of ROS in a state of homeostasis to prevent damage to the structure of DNA, proteins, and lipids. Under adverse conditions, ROS can induce activation of transcription factors such as Nrf2. This activated protein will translocate intranuclearly and then attach to the Antioxidant Response Element (ARE) and leads to the activation of genes that play an important role in the production of antioxidants. Therefore, it is necessary to produce enough antioxidants to prevent damage to cells in the body caused by ROS [57].

Andargie et al. (2021) reported that cell-free DNA (cfDNA) as a biomarker of injury in COVID-19 patients increased to produce in excessive mitochondrial ROS (mtROS) in renal tubular cells via TLR9 [58]. Mitochondrial-derived ROS in nerve cells also have a harmful role in stroke, brain aging, and neurodegenerative disorders [56]. Overproduction of ROS from the infection of SARS-COV2 will deprive the antioxidants system. There will be extensive cell damage if the high levels of ROS are not balanced with the production of antioxidants [59]. DNA damage in infected cells inhibits the expression of the key redox-sensitive transcription factor, Nrf2, that generally provide the primary protective measures of cells against oxidative stress [60].

## 5. Nuclear Factor Erythroid 2 (Nrf2)

Nuclear factor E2-like factor 2 (Nrf2 or NFE2L2) is a member of the cap ‘n’ collar (CNC) subfamily of the basic leucine zipper transcription factor, activated by oxidative stress [61,62,63]. Under physiological conditions, the Nrf2 signaling pathway is regulated by inhibition of Nrf2 protein degradation, which is mediated by Kelch-like ECH-associated protein 1 (Keap1). Nrf2 is maintained in the cytoplasm by Keap1. The primary function of Keap1 is as an adapter for the Cul3/Rbx1) E3 ubiquitin ligase complex. Keap1 binds to the Nrf2 substrate leading to Nrf2 degradation via the 26S proteasome [64]. When cells are under stress, the generation of ROS will cause dissociation of the Nrf2–Keap1 complex. At that time, Nrf2 will move to trigger the transcription of many genes involved in antioxidant responses and redox homeostasis in the nucleus [65]. Meanwhile, in reaction to nitric oxide (NO), Nrf2 is translocated to the nucleus to bind ARE and regulate the intracellular antioxidant activity, maintenance of cellular redox homeostasis, detoxification, affect mitochondrial biogenesis, and glutathione homeostasis, which operates through activation of transcription with a series of genes, including heme oxygenase-1 (HO-1), NAD(P)H (nicotinamide adenine dinucleotide phosphate) quinone oxidoreductase 1 (NQO1), superoxide dismutase (SOD), and glutathione S-transferase (GST) [66,67]. Several in vivo and in vitro studies have demonstrated the importance of this transcription factor in upregulating ARE-mediated gene expression [68,69].

**Figure 4:**
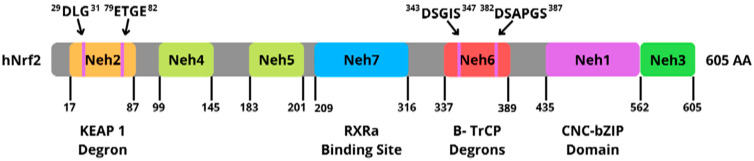
Domain structure of human Nrf2.

The homology domain of Nrf2-ECH (Neh) is a transcription factor that can be mapped into several regions/domains. There are seven known Neh domains and each has a different function; this is shown in Figure 4 [70].

The Neh1 domain is a DNA-binding peptide in *Drosophila melanogaster* and has an important role in heterodimerization and as a transcription factor. The N-terminal Neh2 domain plays a role that is important in Keap1-mediated repression and negatively controls Nrf2 activity. The C-terminal Neh3 domain plays a role in the activation of Nrf2 transcription and target gene transactivation. Neh4 and Neh5 act in the binding of coactivator CREB-binding protein, thereby increasing gene transcription synergistically. The Neh6 domain plays a role in the independent regulation of Keap1–Nrf2 and negatively controls Nrf2. Neh 7 suppresses the activity of Nrf2 by preventing coactivator recruitment to the domain of Neh4 and Neh5. This is because Neh7 has direct protein interactions between Nrf2 and the DNA-binding domain of the retinoid receptor Xa (RXRe).

The levels of plasma Nrf2 showed a positive correlation with Th2 cytokines (IL-4 and IL-13) and a negative correlation with the Th1 cytokine levels (TNF-α and IFN-γ), so the lower Nrf2 levels favored Th1 cytokine production. Several studies have also demonstrated that induction of the Nrf2 pathway by activators such as tertiary butylhydroquinone (tBHQ) in CD4^+^ T cells stimulates the activity of Th2 cytokines (IL-4, IL-5, IL-13) transcription and simultaneously inhibits Th1 cytokine production. Th1/Th2 cell differentiation from CD4^+^ T cells is important for tailoring adaptive immune responses to specific pathogens, thereby allowing flexibility of T cell function and downstream immune activity [71]. In addition, Nrf2-deficient dendritic cells exhibit increased oxidative stress, thereby conferring Th2-like immune responses, leading to an altered Th1 and Th2 balance [72].

Studies have shown that the Nrf2 signaling pathway is widely used as a promising therapeutic candidate for various diseases [72]. In studies in vivo, activation of Nrf2 resulted in decreased oxidative stress and increased bacterial phagocytosis by macrophages [62,63].

## 6. The Keap1–Nrf2 System

**Figure 5:**
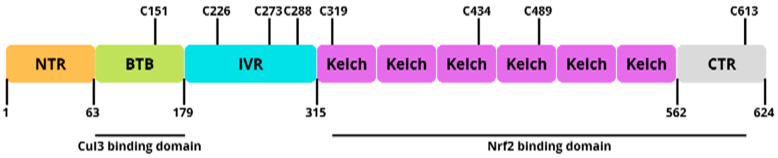
Domain structures and functional domains of Keap1.

Keap1 is a protein rich in cysteine. As shown in Figure 5, there are three main cysteine sensors on Keap1, namely, the C151, C273, and C288 cysteine sensors. This cysteine sensor has the function of inducing Nrf2 into the nucleus and modifying the structure of KEAP1 by electrophiles so the oxidizing agents can carry out target gene expression [73]. There are three main domains in Keap1, namely, the Broad complex/Tramtrack/Bric-a-brac (BTB) domain, Kelch domain, and the intervening region (IVR) domain. The BTB domain binds to CuI3 present at the N-terminal by forming homologs and dimers [74]. The IVR domain connects the Kelch domain and the BTB domain rich in cysteine residues that play an important role in Keap1 activity. There are six Kelch repeat sequences in the Kelch domain that are at the C-terminal domain. It has interactions with the Neh2 domain of Nrf2, which play an important role in the interaction of Keap1 and Nrf2. Keap1 is considered an electrophile and ROS biosensor. It has been reported that Keap1 is an important unit of the Keap1–Nrf2 system, functions to protect against oxidative damage to cells under oxidative stress, and in Nrf2 the activity of Nrf2 [75].

The multifunctional autophagy adapter, p62 (sequestosome 1), has a role in noncanonical Nrf2 activation [76]. p62 is a possible early target for aging intervention. This intervention is possible because it has an interaction with the Keap1–Nrf2 pathway. According to the results of the study, p62 showed that it could exert an anti-aging effect through Keap1–Nrf2 signaling [77].

Signaling by Keap1/Nrf2/p62 (accumulation of p62 and inhibition of Keap1 mediates Nrf2 activation) has an important role in reducing cell senescence accelerated by excessive cellular oxidative stress due to COVID-19 infection [75,78]. In this case, the accumulation of ROS plays an important role in the aging process, which will increase the risk of severe complications in SARS-CoV-2 infection that causes the emergence of long COVID-19 syndrome. The Keap1–Nrf2 signaling pathway in higher organisms is a way to increase longevity [75]. The most important cellular antioxidant system is the Keap1–Nrf2 signaling pathway, which regulates the expression of various antioxidant enzymes [75,79].

## 7. Molecular Crosstalk between Nrf2 and Nuclear Factor Kappa B (NF-κβ) Response Pathways

**Figure 6:**
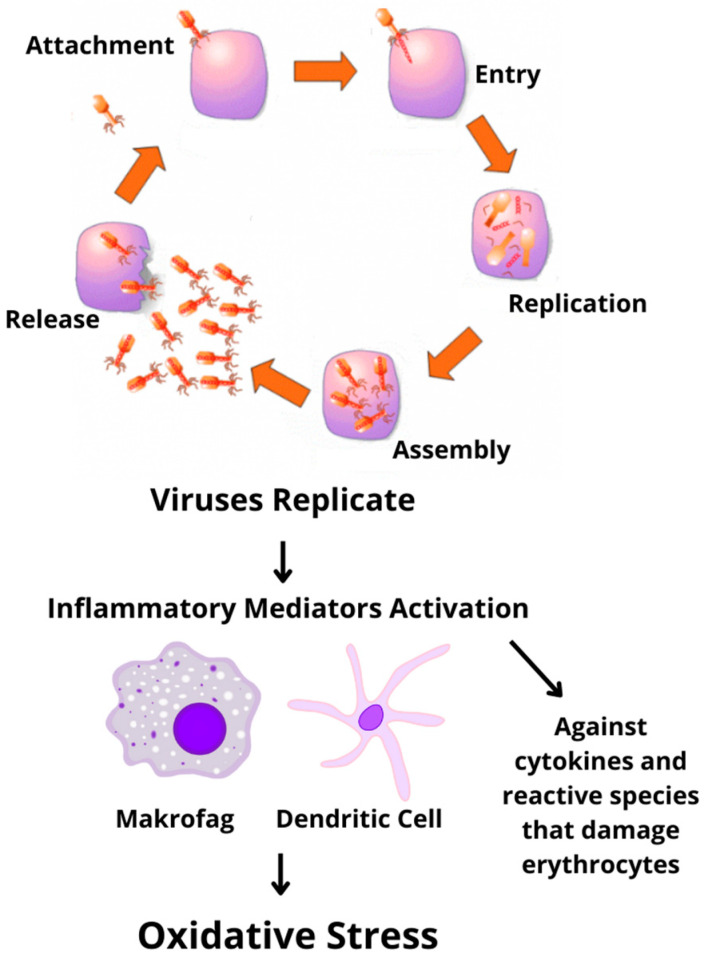
Cytokine expression via the NF-κβ pathway causes a cytokine storm.

**Figure 7:**
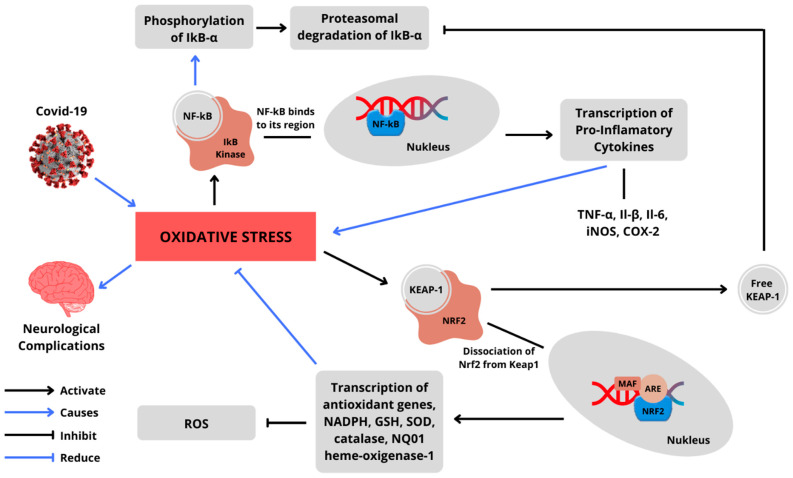
Crosstalk between the Nrf2 and NF-κβ pathways mediated by infection of COVID-19 leads to neurological complications.

Studies have shown the involvement of Nrf2 and NF-κβ in the COVID-19 pathogenesis. As shown in Figure 6, the replicating of viruses can induce oxidative stress that makes a cytokine storm. This is due to the involvement of this signaling pathway in neurological complications of immune injury caused by cytokines. There are studies of transgenic mice that reported that when the Nrf2 pathway is activated and blocked, the response is different. At the activation of the Nrf2 pathway, it suppresses oxidative stress and improves cognitive function in mice. However, when this pathway is blocked, it results in oxidative injury and decreased neuronal viability [80]. The potential mechanism of crosstalk between Nrf2 and NF-κβ is well known, as shown in Figure 7. A natural or synthetic immunomodulator that can inhibit NF-κβ or modulate the Nrf2–Keap1 pathway influencing the crosstalk could be one of the promising candidates in serving the targeted pathology of this pathway. This pool of candidates can be explored as a therapy against COVID-19 [10].

## 8. Natural Antioxidant Therapies for Activating Nrf2

Many claim that nutritional supplements in the form of food or drinks can increase immunity, especially during the COVID-19 pandemic [81]. As it relates to oxidative stress, a person’s nutritional status has a major impact on the immune system and on the development of comorbidities that are considered risk factors for COVID-19 [82]. Phytochemicals are metabolites that have been identified in commonly consumed plants. Based on their chemical structure, phytochemicals are divided into alkaloids, phenols, polyphenols, terpenoids, and compounds containing sulfur [83].

**Figure 8:**
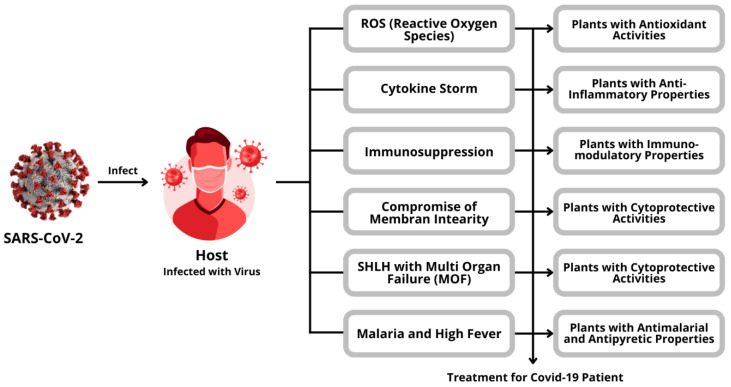
The role of plants in healing COVID-19.

**Figure 9:**
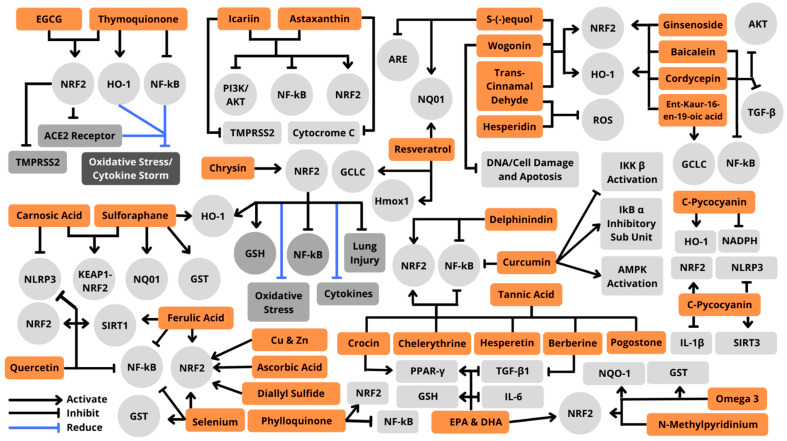
Mechanism of natural antioxidant compounds in Nrf2 activation.

Phytochemicals have various mechanisms (Figure 8), one of which is as an antioxidant agent that donates electrons to stabilize oxidants, hence preventing oxidation of other molecules. At a molecular level, a growing body of evidence indicates a close connection between specific biological responses, such as an increase in ROS, and COVID 19 pathogenesis, which can cause oxidative stress and associated oxidative damage to DNA, lipids, proteins, and other molecules, resulting in the development of several diseases [83]. The complex immune system protects against oxidative damage in the presence of cells that already have an endogenous antioxidant defense system, including various antioxidant enzymes such as superoxide dismutase, catalase, glutathione reductase, and glucose-6-phosphate dehydrogenase [84,85]. Under normal conditions, these endogenous antioxidants maintain a cellular redox state by neutralizing free radicals. However, if endogenous antioxidants are insufficient due to exposure to stressors that increase the production of oxidants, such as the emergence of chronic diseases, pollutants, injury, or exercise can exceed the ability of endogenous cellular antioxidant defenses [86]. Under such circumstances, exogenous phytochemicals, or alternative antioxidants need to be supplemented to the body. For example, phenolic antioxidants, carotenoids, vitamins, and minerals may be required to maintain cellular redox status and balance the oxidation of proteins, lipids, and DNA [87,88]. Several natural compounds used as Nrf2 activators are summarized in Table 1 and Figure 9.

Polyphenols are the most abundant natural antioxidants that can be found in various parts of plants such as roots, stems, flowers, leaves, and pulp [151]. Polyphenols have 10 or more classes, but phenolic acids, flavonoids (flavonols, flavanones, flavan-3-ols, anthocyanidins, isoflavones, and flavones), lignans, and stilbenes are the four main classes [152]. For it to be active in the body, polyphenols undergo various intestinal transformations with the help of digestive enzymes. During transit in various tissues and organs, these compounds can perform antiviral, antibacterial, and antiparasitic functions, among others. For example, activating Nrf2 and transcription of vitagenes suppresses the ROS production and inflammation. With this, polyphenols have the potential to be used as a therapy against SARS-CoV-2 infection [153,154].

Micronutrients, including vitamins and trace elements (copper, zinc, and selenium), are associated with immune function. Deficiency in these micronutrients can cause cell-mediated damage to innate immune cells, changes in cytokine production, and decreased antibodies, resulting in increased susceptibility to various infections, including SARS-Cov-2 [155,156]. Thus, consumption of foods rich in vitamins, selenium, zinc, and copper can have a positive impact on patients from long COVID-19 [82].

Terpenoids are one of the largest classes (>50,000) of the most structurally diverse natural plant compounds and have various biological activities. They are widely used as medicinal agents [157]. The mevalonate pathway (MVA) is a common pathway for the biosynthesis of terpenoids in higher plants, fungi, and animals. Taxol [158], astaxanthin [99], carnosic acid [107], crocin [113,122], ginsenoside [159], and pogostone [135] are some examples of terpenoid compounds capable of activating Nrf2, and therefore has the potential as a long-term treatment for COVID-19.

Alkaloids are secondary metabolites of plants (20% species) containing nitrogen atoms in heterocyclic rings. Alkaloids from various plant families, such as *Annonaceae*, *Berberidaceae*, *Menispermaceae*, *Papaveraceae*, *Ranunculaceae*, *Rutaceae*, and *Chelidonium* have been widely used in traditional and modern medicines [160,161]. For example, berberine and chelerythrine were reported to be able to activate Nrf2 [101].

Phycobiliproteins have various bonds between pigments and proteins that are influenced by the environment. It is divided into three types, namely, phycoerythrin (phycoerythrobilin complex and protein which is red), phycocyanin (phycocyanobilin complex and protein is blue in color), and indigo-blue allophycocyanin (phycocyanobilin complex and protein with different absorption spectrum). Phycobiliproteins have antioxidant properties because they can control oxidative stress by regulating H2O2-induced *p*-Nrf2/SOD expression [102].

An adenosine nucleotide compound, cordycepin, was reported to exert a protective effect against inflammatory injury for many diseases, including asthma, atherosclerosis, atopic dermatitis, hepatitis, Parkinson’s disease, and rheumatoid arthritis. This compound can regulate the signaling pathways for NF-κB, RIP2/Capase-1, Akt/GSK-3β/p70S6K, TGF-β/Smads, and Nrf2/HO-1. In addition, cordycepin can also increase immunity, inhibit viral RNA proliferation, and suppress cytokine storms, and hence can potentially be used as a treatment for viral infections, especially COVID-19 [112,162].

A study of MRC-5 lung cells in mice reported that one of the sulfide compounds in garlic, namely, diallyl sulfide [163], can modulate Nrf2. It can increase the activity of glutathione peroxidase, glutathione reductase, glutathione S-transferase, NQO1 pulmonary superoxide dismutase, GSH/GSSG ratio, and mRNA catalase. This was demonstrated by an increase in the total pulmonary Nrf2 and nuclear Nrf2 translocations in the lungs of diallyl sulfide-treated mice compared to the untreated (*p* < 0.05). From this, diallyl sulfide can be used as a dietary preventive agent against oxidative stress-induced lung injury [163].

Omega-3 polyunsaturated fatty acids (DHA, DPA, and EPA) are abundant in fish oil and are known to be anti-inflammatory in heart disease [164,165,166]. This fatty acid can replace arachidonic acid in the body when consumed, so it can help the immunological process and reduce the production of inflammatory mediators (eicosanoids, adhesion molecules, cytokines, and certain enzymes) [165]. In addition, because of its role as an Nrf2 activator, it is considered a treatment for severe cases of long COVID-19 due to excessive inflammation [120,121].

In a study of human colon carcinoma (HT29) cells, it was found that the pyridinium derivative N-methylpyridinium ion (NMP) in roasted coffee can act as a strong activator of Nrf2 and ARE-dependent gene expression. This impact on Nrf2 signaling is determined by the substitution pattern in the pyridinium core structure [93].

Isothiocyanates are chemical compounds formed from glucosinolates. Sulforaphane, which is an isothiocyanate, can be formed in plant tissue and the mammalian microbiome through the action of myrosinase, β-thioglucosidase. In clinical trials, sulforaphane has been shown to have a role as an NRF2 activator against the COVID-19 virus [167,168].

One of the quinone compounds, thymoquinone, can activate Nrf2 by phosphorylation, which causes translocation in the nucleus and Nrf2 binds to ARE and Maf. This binding resulted in the reduction of NF-kB, inflammation, cytokine production, and oxidative damage. In addition, there was also an increase in the detoxification of cytoprotective genes and the HO-1 enzyme. Thymoquinone also decreases the expression of GRP78 and the ACE2 receptor, thereby reducing viral entry. This shows that thymoquinone has the potential to reduce SARS-CoV-2 infection [169].

## 9. Molecular Mechanism of Natural Antioxidant to Activate Nrf2: Prediction and Prospect

A preliminary study on the molecular mechanism of natural compounds against oxidative stress was reported using in silico research methods. Li et al. (2019) clarified the interaction capabilities of 178 natural antioxidants to activate the Nrf2 binding site in Keap1 and activating the Nrf2-ARE signaling pathway using molecular docking and 3D−QSAR methods. The results showed that 24 of 178 natural antioxidants can effectively inhibit Keap1–Nrf2 interactions. The structure of these compounds is rich in oxygen or glycosides suggesting that oxygen-rich compounds or glycosides provide an effective Nrf2 activation effect [170]. In addition, a study using molecular dynamics on 50 natural antioxidants, reported that 3 of the 50 compounds showed high binding affinity to the Keap1 Kelch pocket. This could be due to the hydrogen-bonding factor by the Val418 residue as an addition to protein–ligand binding, as well as the electrostatic/hydrophobic interactions formed by the Ala366 residue. Furthermore, three compounds, including resveratrol (binding energy value −7.8 kJ/mol), can be used as drug candidates with therapeutic functions against oxidative stress-mediated diseases due to their ability to activate Nrf2 by inhibiting Keap1 [171]. Based on in vitro results, EGCG abolishes the interaction between Keap1 and Nrf2 and activates Nrf2 in the cytosol [172]. Sun et al. found that EGCG inactivates the KEAP 1 protein, thus mediating the function of EGCG in activating NRF2 [172].

**Figure 10:**
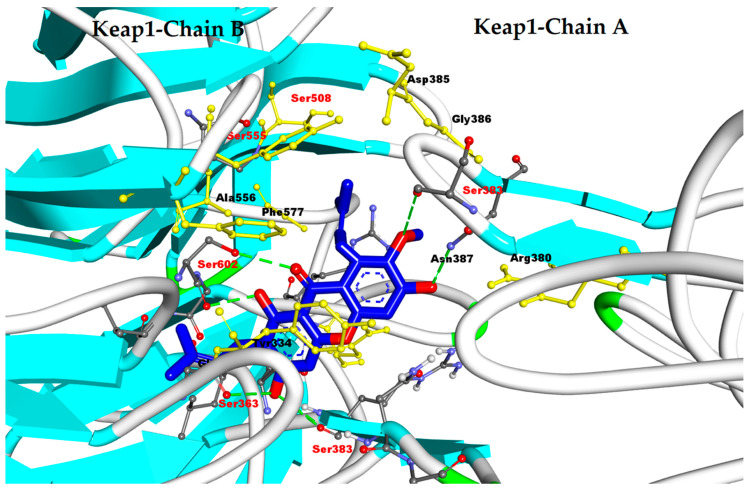
Interactions of alpha-mangsotin (AM) against Keap1 to activate Nrf2 (PDB code: 4l7b) (yellow atoms of amino acid: hydrophobic interactions; green dotted line: hydrogen bond interaction).

Here, we simulated four natural product compounds, including epigallocatechin gallate (EGCG), kaempferol, apigenin, resveratrol, and alpha mangostin (AM), using a 100-run genetic algorithm of molecular docking simulation (Autodock 4.2) against Keap1−Nrf2 (PDB code 4l7b) [173]. From previous studies, these compounds have strong antioxidant activity against ROS [174,175,176,177,178]. From the results, AM, kaempferol, EGCG, apigenin, and resveratrol have free-energy bindings of −7.67, −6.73, −6.64, −6.24, and −6.00 kcal/mol, respectively. Interestingly, AM obtained good interaction against the Keap1 binding pocket (free energy binding: −7.73 kcal/mol). The strong hydrogen bond is formed with Ser602, Ser338, and Asn387 and hydrophobic interaction with Ser363, Ala556, Ser555, Ser508, and Phe577, as shown Figure 10. However, effort is needed to demonstrate in vitro that AM upregulates NRF2 to prevent ROS by inactivating Keap1.

**Figure 11:**
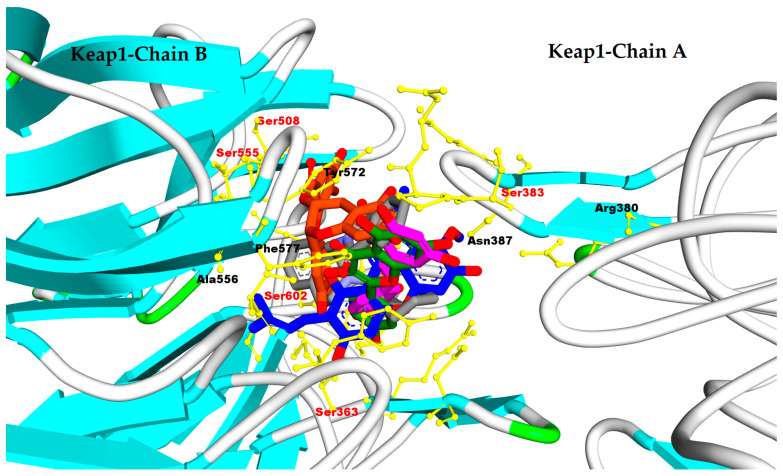
Pose of AM, kaempferol, EGCG, apigenin, and resveratrol against the binding site of Keap1. (Grey carbon: native ligand (green carbon: native ligand, blue carbon: AM, green carbon: kaempferol, pink carbon: resveratrol, and orange carbon: EGCG, yellow atoms: amino acid residues that have interactions with hydrophobic moiety of ligands).

Overall, the four compounds interact with the conserved amino acid residues in the Keap1-binding pocket, such as Ser602, Ser363, Ser508, and Ser555 (as shown Figure 11). These amino acid residues have been reported to be crucial in the interface structure of Keap1−Nrf2; thus, the results indicate that a high binding affinity with Keap1 might directly inhibit Keap1−Nrf2 protein−protein interaction as Nrf2 activators. These compounds are capable of activating Nrf2 and therefore have the potential to be used as long-term treatment of COVID-19 [179].

## 10. Conclusions

Long COVID is a syndrome that occurs for weeks or months after COVID-19 infections. To date, no cure has been reported to minimize the symptoms of long COVID. From the literature search, it is evident that natural antioxidants play an important role in Nrf2 activation and NF-kβ suppression, indicating a relationship with the pathogenesis of COVID-19. Natural antioxidants from several sources have been shown to prevent oxidative stress and hence have the potential to prevent and treat COVID-19 disease, especially the long COVID-19 syndrome in humans. Patients with COVID-19 having complications such as long COVID-19 can consume nutrient-dense foods, fortified and enriched foods, or supplements that contain natural antioxidants. However, further research is required, especially in determining the dose for optimal effect, as well as identifying potential side effects.

## Figures and Tables

**Table 1:** The bioactive compounds reported in the literature that can activate Nrf2.

Natural Antioxidant	Group	Sources	Mechanism	Reference
(−)-epigallocatechin-3-gallate (EGCG)	Polyphenol	*Camellia sinensis*	Activation of nuclear factor-erythroid-2 related factor 2 (Nrf2) suppresses angiotensin converting enzyme 2 (ACE2) and transmembrane protease serine 2 (TMPRSS2)	[89,90,91,92]
5-O-caffeoylquinic acid (CGA)	Polyphenol	*Coffea* sp.	Nrf2 translocation activator and ARE-dependent gene expression (NQO-1 and (GST)A1)	[93,94]
Ascorbic acid	Vitamins	Oranges, lemons, strawberries, broccoli, red peppers, mangoes	Nrf2 driven by ARE	[95,96]
Astaxanthin	Terpenoid	*Haematococcus pluvialis*, *Xanthophyllomyces dendrorhous*	Modulation of Nrf2/ARE signaling, inhibits cytochrome c (Cyt c), regulates phosphoinositide-3-kinase–protein kinase B/Akt (PI3K-PKB/Akt) pathway, modulation of Nrf2/nuclear factor-kappaB (NF-κβ) signaling pathways	[97,98,99]
Baicalein	Polyphenol	*Scutellaria baicalensis*	Increases Nrf2/heme oxygenase-1 (HMOX1, HO-1) cascade; inhibits NF-κβ activation	[100]
Berberine	Alkaloid	*Annonaceae*, *Berberidaceae*, *Menispermaceae*, *Papaveraceae*, *Ranunculaceae*, *Rutaceae*	Nrf2 activation, NF-κβ suppression and transforming growth factor (TGF)-β1-mediated fibrotic events	[101]
C-Phycocyanin	Phycobiliprotein	*Cyanobacterium Spirulina*	AhR agonists and inhibition of Nicotinamide Adenine Dinucleotide Phosphate (NADPH) oxidase activity promote transcription of genes encoding Nrf-2, upregulate HO-1	[102,103]
Calcitriol	Vitamins	UVB light	Nrf2 is activated when vitamin D receptor (VDR) binds to retinoic acid-related receptor (RXR); Nrf2 activation inhibition of reactive oxygen species (ROS)- NLR family pyrin domain containing 3 (NLRP3)- interleukin (IL)-1β signaling; Nrf2 interaction with peroxisome proliferator activated γ receptor coactivator (PGC-1α) regulates Sirtuin (SIRT) 3 expression	[104,105,106]
Carnosic acid	Terpenoid	*Rosmarinus officinalis*	Activation of the Kelch-like ECH-associated protein 1 (Keap1)/Nrf2 transcriptional pathway, inhibition of the NLRP3 inflammasome	[107,108]
Chelerythrine	Alkaloid	*Chelidonium*	Nrf2 activation reduced nuclear translocation of NF-κβ p65	[109]
Chrysin	Polyphenol	*Carthamus tinctorius*	Activates NF-κβ, activates the SIRT1/Nrf2 pathway	[110]
Copper (Cu) and Zinc (Zn)	Trace element	*-*	Nrf2/ARE activates superoxide dismutase (SOD) transcription	[111]
Cordycepin	Adenosine nucleotide	*Cordyceps militaris*	Regulate signaling pathways Nrf2/HO-1, NF-κβ, Akt/Glycogen Synthase Kinase 3β (GSK-3β)/p70S6K, RIP2/Caspase-1, TGF-β/Smads	[112]
Crocin	Terpenoid	*Crocus sativus*	Downregulates NF-κβ, upregulates Peroxisome Proliferator Activated Receptor-γ (PPAR-γ) and Nrf2 expression	[113]
Curcumin	Polyphenol	*Curcuma longa*	Enhances biologic effects of Nrf2 through interaction with Cys151 in Keap1; activates NLRP3 by triggering the SIRT1/Nrf2 pathway to elicit downstream cytokines (IL-1β, IL-18, IL-6, and TNF-α)	[114,115,116,117,118]
Delphinidin	Polyphenol	*Aristotelia chilensis*	Regulating Nrf2/NF-κβ tissue, inducing intact autophagy	[119]
Diallyl sulfide	Sulfide	*Allium sativum*	Induction of Nrf2 nuclear translocation via the ERK/p38 signaling pathway	[34]
Eicosapentaenoic acid (EPA) and docosahexaenoic acid (DHA)	Omega-3 FA	Fresh oily fish, microalgae, marine protists, dinoflagellates, cereals, grains, legumes, and some fruits/vegetables	Upregulation of NRF2, reduces isoprostane F2, induces PPARγ, modulates toll-like receptor 4 (TLR4) reduced phosphorylation, NF-κβ, IL-6, Tumor Necrosis Factor (TNF)α, TGFβ; induce mitogen activated protein kinase (MAPK) phosphatase and increase glutathione (GSH)	[120,121]
Ent -kaur-16-en-19-oic acid	Terpenoid	*Aralia Continentalis*	Activates Nrf2 and induces expression of Nrf2-regulated genes (Glutamate-Cysteine Ligase Catalytic (GCLC) and HO-1) without affecting NF-κβ	[122]
Ferulic acid	Polyphenol	*Pinus maritima*	Increase SIRT1 expression, SIRT1 enhances phase 2 induction of antioxidant enzymes via Nrf2 and inhibition of NF-κβ	[123]
Ginsenoside	Terpenoid	*Panax ginseng*	Activate the Nrf2/HO-1 pathway	[124]
Hesperidin	Polyphenol	*Citrus sinensis*	Upregulated Nrf2, reduced ROS, increased glyoxalase 1 (Glo-1)	[125,126,127,128]
Hesperetin	Polyphenol	*Isatis indigotica*	Enhances antioxidant activity via the ERK/Nrf2-regulated signaling kinase pathway, high affinity for the protease site of the ACE2 receptor	[129,130]
Icariin	Polyphenol	*Herba Epimedii*	Activation of Nrf2-heme oxygenase 1 (HO-1) through increased expression of Nrf2 and decreased expression of NF-κβ, suppresses ROS generation and increases glutathione levels	[131]
N-methylpyridinium	Pyridinium	*Coffea* sp.	Nrf2 translocation activator and ARE-dependent gene expression ((NADPH), quinone oxidoreductase 1 (NQO1) and glutathione s-transferase (GST) A1)	[93,94]
Quercetin	Polyphenol	*Curcuma domestica valenton*, *Cuscuta reflexa*, *Emblica officinalis*, *Foeniculum vulgare*, *Mangifera indica*, *Santalum album*, *Withania somnifera*	Suppression of NLRP3 activates Nrf2, SIRT1 and Thioredoxin-interacting protein (TXNIP)	[132,133,134]
Pogostone	Terpenoid	*Pogostemon cablin*	Increases Nrf2-dependent genes (NQO-1, GCLC, HO-1), suppresses NF-κβ-regulated genes (IL-1 β, IL-6, TNF-α)	[135]
Resveratrol	Polyphenol	*Veratrum grandiflorum*, *Polygonum cuspidatum*, *Vitis vinifera*, *Arachis hypogaea*, *Morus rubra*	Binding of Nrf2 with ARE activates NQO1, GCLC, and HMOX1	[136,137,138,139]
S- (-) equol	Polyphenol	*Glycine max*	Increase Nrf2 and HO-1 and NQO1, interfere with HA-Nrf2 nuclear translocation, decrease ARE-luciferase activity	[140]
Selenium (Se)	Trace element	*-*	Up-regulation of Nrf2 signaling, increased glutathione synthesis, downregulation of NF-κβ pathway	[141]
Sulforaphane	Isothiocyanate	*Brassica oleracea*	Activation of the Nrf2-Keap1 signaling pathway induces glutathione S-transferase, HO-1, NQO1, and ‘Uridin 5′-difosfo-glukuronosyltransferase’ (UDP-glukuronosiltransferase, UGT)	[142,143,144,145]
Tannic acid	Polyphenol	Green tea, fruits, cereals, red wine	Modulated by NF-κβ and NRF2 pathways reduction IL-6, IL-8, TNF-α	[34]
Thymoquinone	Quinone	*Nigella sativa*	Activates Nrf2 which decreases ACE2 expression and inhibits NF-κβ	[146,147]
Trans-cinnamaldehyde	Polyphenol	*Cinnamomum cassia*	Blocking abnormal accumulation of ROS, activation of the Nrf2/HO-1 signaling pathway	[148]
Phylloquinone	Vitamins	Collards, turnip, broccoli, spinach, kale, dried prunes, kiwifruit, avocado, blueberries, blackberries, grapes, pine nuts, cashews, pistachios	Activation of gamma-carboxyglutamic acid (Gla) protein decreased NF-κβ phosphorylation, Monocyte Chemotactic Protein-1 (MCP-1) secretion, increased Nrf2 expression	[149]
Wogonin	Polyphenol	*Scutellaria baicalensis Georgia*	Activates the Nrf2/HO-1 signaling pathway to inhibit DNA, cell damage and apoptosis	[150]

## Data Availability

Data is contained within the article.
